# Complement C3 overexpression activates JAK2/STAT3 pathway and correlates with gastric cancer progression

**DOI:** 10.1186/s13046-019-1514-3

**Published:** 2020-01-13

**Authors:** Kaitao Yuan, Jinning Ye, Zhenguo Liu, Yufeng Ren, Weiling He, Jianbo Xu, Yulong He, Yujie Yuan

**Affiliations:** 1 Center of Gastrointestinal Surgery, The First Affiliated Hospital, Sun Yat-sen University, Guangzhou, People’s Republic of China; 2 Center of Gastric cancer, Sun Yat-sen University, Guangzhou, People’s Republic of China; 3 Department of Thoracic Surgery, The First Affiliated Hospital, Sun Yat-sen University, Guangzhou, People’s Republic of China; 4 Department of Radiation Oncology, The First Affiliated Hospital, Sun Yat-sen University, Guangzhou, People’s Republic of China

**Keywords:** Complement, Gastric cancer, Immune signature, Tumor stage, STAT3, Prognosis prediction

## Abstract

**Background:**

Localized C3 deposition is a well-known factor of inflammation. However, its role in oncoprogression of gastric cancer (GC) remains obscured. This study aims to explore the prognostic value of C3 deposition and to elucidate the mechanism of C3-related oncoprogression for GC.

**Methods:**

From August to December 2013, 106 GC patients were prospectively included. The regional expression of C3 and other effectors in gastric tissues were detected by WB, IHC, qRT-PCR and other tests. The correlation of localized C3 deposition and oncologic outcomes was determined by 5-year survival significance. Human GC and normal epithelial cell lines were employed to detect a relationship between C3 and STAT3 signaling pathway in vitro experiments.

**Results:**

C3 and C3a expression were markedly enhanced in GC tissues at both mRNA and protein levels compared with those in paired nontumorous tissues. According to IHC C3 score, 65 (61.3%) and 41 (38.7%) patients had high and low C3 deposition, respectively. C3 deposition was negatively correlated with plasma levels of C3 and C3a (both *P <* 0.001) and positively correlated with pathological T and TNM stages (both *P <* 0.001). High C3 deposition was identified as an independent prognostic factor of poor 5-year overall survival (*P* = 0.045). In vitro C3 administration remarkably enhanced p-JAK2/p-STAT3 expression in GC cell lines but caused a reduction of such activation when pre-incubated with a C3 blocker. Importantly, C3 failed to activate such signaling in cells pre-treated with a JAK2 inhibitor.

**Conclusions:**

Localized C3 deposition in the tumor microenvironment is a relevant immune signature for predicting prognosis of GC. It may aberrantly activate JAK2/STAT3 pathway allowing oncoprogression.

**Trial registration:**

ClinicalTrials.gov, NCT02425930, Registered 1st August 2013.

## Background

Gastric cancer (GC) is the second leading cause of cancer-associated death and the fifth most common malignancy worldwide [1]. To date, GC remains an excessive health burden in several Asian countries, especially in China, Korea and Japan [2]. In spite of recent progress in surgical and comprehensive therapies, improvement in oncologic outcomes of the advanced GC population is limited [3]. As a result, it has been a focus to explore the mechanisms essential for the development and progression of GC [4].

Complement is a phylogenetically conserved branch of the innate immune system. It is traditionally regarded as a network of proteins that rapidly respond to microbial intruders, triggering the release of inflammatory mediators, phagocytic responses and cellular lysis [5]. Growing evidence has pointed to a fascinating paradigm shift: complement activation within the tumor microenvironment could serve a tumor-promoting role by perpetuating local T cell immunosuppression and chronic inflammation, which eventually promotes tumor immune escape, outgrowth and distant metastasis [6–9]. Complement-derived effectors, such as C3a, C3b and C5a, and downstream signaling molecules have been implicated in processes ranging from tumor cell anchorage and proliferation to tumor-related angiogenesis, matrix remodeling, migration, tissue invasiveness and metastasis [10–13].

Inflammatory cytokines, including TNF-α, IL-6 and IL-22, could activate Janus kinase 2/signal transducers and activators of transcription (JAK2/STAT3) pathway in GC cells [14, 15]; consequently those effectors were possibly contributed to local inflammation through complement activation. Emerging evidence has suggested that regional deposition of complement components played an essential role in local inflammation and progression of cancer cells of various histological origin, such as cervical cancer, lung cancer, thyroid cancer and colorectal cancer [6, 16–18].

We designed this study to investigate the regional deposition of C3 and its effectors, and to detect the relationship between such deposition and tumor progression with clinical and laboratory GC.

## Methods

### Bioinformatic analysis

Complement gene expression in GC by RNA sequencing were retrieved from the UCSC Cancer Genomics Browser, which were collected from The Cancer Genome Atlas (TCGA) data portal. 384 GC tissues and 37 tumor-adjacent normal tissues were harvested from the TCGA cohort. Additional 12 samples with paired tissues were extracted from the Oncomine database to assess gene and protein expression of the complement system for GC.

### Patients and samples

From August to December 2013, adult patients with confirmed diagnosis of GC were prospectively enrolled. Consecutive participants referred or admitted to our center for surgical treatment were screened for eligibility. All included patients were managed and followed up as our published procedures [2, 19]. Inclusion criteria were: (1) A primary GC tumor should be resectable on the basis of preoperative evaluation, with no suspicion of distant metastasis; (2) adult age between 18 and 75 years, with no limitation of gender; (3) A radical gastrectomy with adequate lymphadenectomy was suggested after a multidisciplinary team meeting, with adjuvant chemotherapy scheduled or not.

Exclusion criteria included: (1) another synchronized malignancy, severe concomitant basic disease (cardiopulmonary dysfunction, tuberculosis, Crohn’s disease or psychosis) and uncontrolled infection except for Hp infection; (2) requirement of emergency surgery due to tumor progression, history of major abdominal surgery within the last six months; (3) long-term use of corticosteroids, insulin, oral antidiabetic drugs, or other agents for obesity; (4) history of blood transfusion or purification therapy within the last three months.

This study was carried out in accordance with the recommendations of NCCN guidelines for gastric cancer, with written informed consent from all subjects. All subjects gave written informed consent in accordance with the Declaration of Helsinki. The protocol was approved by the Institutional Review Board of First Affiliated Hospital of Sun Yat-sen University and registered at ClinicalTrials.gov (NCT02425930). The last follow-up date was 7th July 2018.

Fresh-paired samples, including tumor and adjacent normal tissues, were collected from specimens isolated in the theatre. Those samples were immediately frozen with liquid nitrogen and stored at − 80 °C for future tests. Peripheral blood samples were routinely collected at baseline, one day before and three days after gastrectomy, respectively. Plasma was obtained by centrifugation (3000 *g*, 20 min, 4 °C) and stored at − 80 °C until tested.

### Cell lines and cell culture

Human SGC-7901 and MGC-803 cells, normal gastric epithelial cells (GES-1) were purchased from the Cell Bank of Chinese Academy of Medical Science (Shanghai, China). All cells were cultured in RPMI-1640 medium supplied with 10% fetal bovine serum (FBS, Thermo Fisher Scientific, Waltham, MA), penicillin (100 U/mL), and streptomycin (100 mg/mL) at 37 °C in a humidified atmosphere of 5% CO_2_. Cells were routinely tested for mycoplasma contamination (MycoAlert™ PLUS Mycoplasma Detection Kit, Lonza). For cell culture, 50–60% confluent cells were transiently incubated with specific agents for 48 h until the extraction of RNA and protein lysates.

Purified recombinant human C3 protein (HuC3, 20 ng/ml, MBS230377, MyBioSource, San Diego, CA) was added to or left out of the culturing medium. Exogenous C3 was depleted with cobra venom factor (CVF; Heng Fei biological technology, Shanghai, China), as previously described [20]. Complement receptor 1 (CR1/CD35, MBS717740, MyBioSource) was used to block C3 activation as previously confirmed [21], with JAK2 blocker (AG490, 25 μM, InvivoGen, Hongkong) used to inhibit STAT3 signaling pathway [22].

### Western blotting (WB) and quantitative real-time polymerase chain reaction (qRT-PCR) analyses

Cell lysates were extracted from gastric tissues and cancer cell lines. The primary antibodies targeted C3, C3a, C5a, CD35, Factor B (fB), IL-6, JAK2, STAT3, pSTAT3 and GAPDH proteins (Abcam, USA). Total protein was obtained through a cell lysis buffer (KeyGene, Nanjing, China) and the protein concentration was quantified using the enhanced BCA protein assay kit (KeyGene, Nanjing, China). The PageRuler™ prestained protein ladder (#26616, Thermo Fisher Scientific, USA) was used to estimate protein sizes. Proteins were separated by 8–10% sodium dodecyl sulfate polyacrylamide gel electrophoresis and electrotransferred to polyvinylidene fluoride (PVDF) membranes (BioRad, Richmond, CA). After that, the membranes with deposited proteins were blocked for 1 h in tris-buffered saline Tween (TBST; T8060, Solarbio) and probed with various primary antibodies, overnight at 4 °C, followed by incubation with rabbit and mouse radish peroxidase-coupled secondary antibody (BA1055, 1:2500; Biosterbio, Wuhan, China) for 1 h. Protein bands were visualized by using an enhanced ECL™ detection kit (KGP1121, KeyGene, Nanjing, China) and captured with a camera (Canon Inc., Japan).

qRT-PCR experiments were performed to detect mRNA expression of C3, C3a and C5, as previously reported [23, 24]. Briefly, total RNA was extracted from GC cell lines using the TRIzol reagent (Invitrogen, Carlsbad, CA, USA). Aliquots of total RNA were reversely transcribed into single-strand-cDNA by incubation with virus reverse transcriptase (6110A; Takara Biochemicals, Kusatsu, Japan). After that, the specific primers for C3, C3a and C5 mRNAs (Additional file 1: Table S1) were used to guide the amplification of cDNA products with 40 cycles at 95 °C for 20s and 60 °C for 1 min. The abundance of each target mRNA was normalized to glyceraldehyde 3-phosphate dehydrogenase (GAPDH) mRNA and presented as 2[(Ct/GAPDH - Ct/gene of interest)].

### Immunohistochemical (IHC) staining

IHC method was employed to detect regional deposition of complement components, including C3 (ab200999, Abcam), C3a (neo-epitope, ab11873, Abcam), C5a (neo-epitope, ab11878, Abcam), fB (ab192577, Abcam), and CR1 (anti-CD35, ab25, Abcam). IHC results were analyzed by two experienced pathologists blinded to patient’s information, and were scored by a semi-quantitative method in which staining of more than 10% of tumor cells was considered positive. The staining intensity was scaled as 0 for negative, 1 for weak (10~40%), 2 for moderate (40~70%) and 3 for strong (> 70%). The average score of staining intensity was calculated with five independent high-power fields using IMAGE PLUS software (Version 6.0, Media Cybernetics, USA). Low and high C3 deposition were defined as ≤1 and > 1 point, respectively. Deparaffinized sections from harvested human tissues were pretreated with 10 mM sodium citrate buffer (pH 6.0, boiling temperature, 30 min), blocked in normal serum (Vectastain ABC Kit; Vector Lab., Inc., CA, USA), incubated with primary antibodies (solution with saline, 1:100) at 4 °C overnight, rinsed and incubated with secondary antibody (EliVision plus, DAB Kit, 9902).

### Immunofluorescence and confocal (IFC) analysis and enzyme-linked immunosorbent assay (ELISA)

Double-marker immunofluorescence staining on paraffin-embedded human tissues were performed as previously described [25]. The primary and secondary antibodies included rabbit anti-human C3 (1:2000, ab20099, Abcam), rabbit anti-human C5a (1:2000, ab11876, Abcam), rabbit anti-phosphorylated (p)-STAT3 (1:2000, Cell Signaling Technology, Danvers, MA, USA) and goat anti-human IL-6 (1:800, R&D systems, Minneapolis, MN). Bound antibodies were revealed by fluorochrome-conjugated antibodies: Alexa Fluor 594 goat anti-Rabbit IgG (H + L; 1:300, ZF-0513) and Alexa Fluor 488 goat anti-Rabbit IgG (H + L; 1:300, ZF-0512) both from Invitrogen Molecular Probes (Carlsbad, CA). All slides were counterstained with DAPI Nucleic Acid Stain (1:1000, Carlsbad, CA) for 60 min. Finally, confocal analysis was performed with a Nikon C2 confocal system (Nikon, Melville, NY) to capture separated and merged images from all sections.

Plasma levels of C3a, C5a and fB in GC patients at perioperative period were monitored using specific ELISA Kits (Thermo Scientific, Frederick, USA). Briefly, 100 μl per well of plasma with standard solution were added to antibody-coated 96-well plates and incubated for 2 h at room temperature, followed by incubation of polyclonal antibody for specific effector for 1 h. Then the plate was washed and incubated with avidin conjugated to horseradish peroxidase (Lifespan BioScience, USA) for 1 h, followed by optical density detection with an ELISA plate-reader at 450 nm. Assays detected only cleaved C3a and C5a peptides in plasma.

### Tumor invasion and migration detection and flow cytometric analysis

A transwell assay system (Corning co. Ltd., USA) was employed to assess GC cell invasion, with a wound-healing assay used to assess tumor migration, as previously published [24, 26].. Nearly 2.0 × 10^4^ cells in 100 μl of serum-free medium were added to each upper chamber for 24 h, with 5 replicate wells set up for each condition. Medium containing 10% FBS applied to the lower chamber as chemo-attractant. After 24 h of incubation, the cells that migrated and adhered to the surface of lower chamber were fixed with ethanol, stained with 0.5% crystal violet, photographed at 200x, and counted at 400x magnification (Olympus, Japan).

A wound-healing assay was performed to assess cancer cell migration. In brief, cells were plated (2 × 10^5^/well) in 6-well plates and grown to 90% confluency. Central bands were artificially scratched with a sterile pipette tip to make a 1 cm wide ribbon. Afterward, the dislodged cells were removed by two PBS washes and serum-free culture medium was added for 48 h. The wound width and cell density were observed at 12 h, 24 h and 48 h, respectively.

Flow cytometric analysis detected the apoptosis rate and cell cycle of GC cells, as previously described [27, 28]. Cancer cells were harvested after 48 h of culture via ethylene diamine tetra-acetic acid-free trypsinization. Human purified C3 protein (20 ng/ml) or CVF protein (40 ng/ml) was selectively added into the culture medium, with PBS added as normal control (NC). Early apoptosis rate was detected using an annexin V-fluorescein isothiocyanate apoptosis detection kit (Oncogene Research, Boston, MA). The cell cycle was investigated via PI/RNase staining methods by using FACScan and CellQuest software (Becton Dickinson, CA).

### Statistical analysis

The relationship between regional expression of C3 and clinical characteristics was analyzed with the *chi*-square test. Continuous variables were compared between both groups with the *t* test. Correlation between C3 deposition and other factors was revealed with a linear regression. Survival analyses were performed using the Kaplan-Meier estimate. The prognostic value of selective parameters was determined with the receiver operating characteristic (ROC) curve analysis, with a value of area under the curve (AUC) approaching 1.0 showing predictive power. All data were analyzed with SPSS® (Version 23.0). Statistical significance was set at 0.05.

## Results

### Patients characteristics

**Fig. 1:**
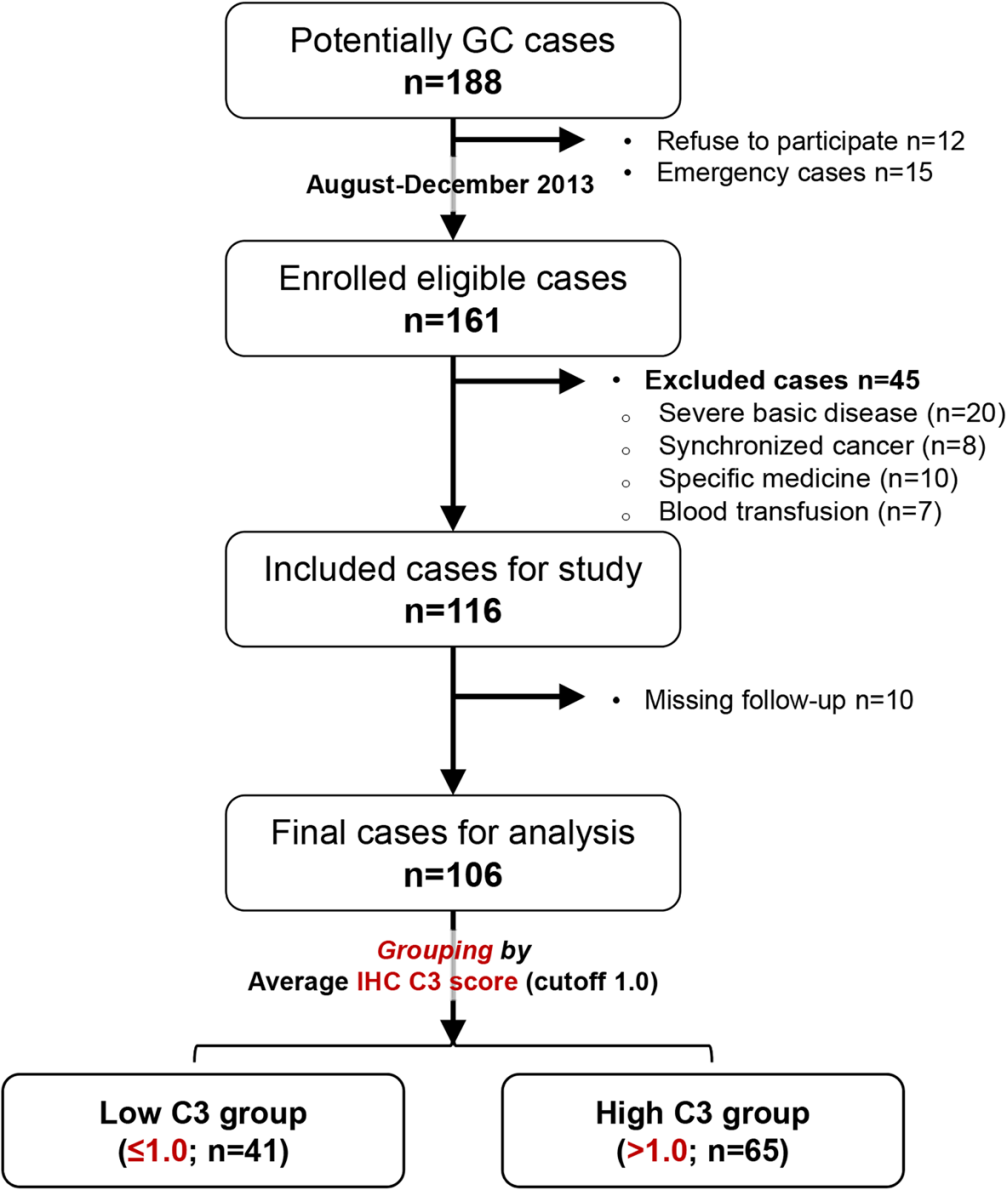
The flow chart of clinical study. Patients qualified with our study criteria were prospectively enrolled, with written informed consent obtained before any treatment. Included patients were allocated to two groups based on the average IHC C3 score

A total of 106 patients were analyzed, with 65(61.3%) males and 41(38.7%) females. The flow chart of study design is shown in Fig. 1. Briefly, 41(38.7%) and 65(61.3%) patients were assigned to low and high C3 deposition groups, respectively. The median follow-up period was 41 (range, 1–57) months, which was significantly shortened in the high C3 group compared with the low C3 group (29 months vs. 43 months, *P =* 0.006). The demographic and baseline characteristics (Table 1) were almost comparable between the two groups (*P* > 0.05), except plasma levels of C3 and C4, and tumor histology (*P <* 0.05). Open gastrectomy plus adequate lymphadenectomy was performed in 98 patients (92.5%), with laparoscopic approach applied in only eight patients (7.5%). The surgical parameters were similar between both groups (Additional file 2: Table S2).

**Table 1:** The demographic and baseline characteristics of patients with gastric cancer

	The Pooled (*n* = 106)	Low C3 group (*n* = 41)	High C3 group (*n* = 65)	*P*-value
Age, yrs	56.4 ± 12.2	59.3 ± 10.5	54.6 ± 13.0	0.056
≤ 65	82 (77.4)	31(75.6)	51(78.5)	0.813
> 65	24 (22.6)	10(24.4)	14(21.5)	
Sex				0.307
Male	65(61.3)	28(68.3)	37(56.9)	
Female	41(38.7)	13(31.7)	28(43.1)	
BMI, kg/m^2^	21.6 ± 3.3	21.9 ± 3.6	21.4 ± 3.0	0.454
Comorbidity				
HTN	9 (8.5)	5(12.2)	4(6.2)	0.303
DM	4 (3.8)	0	4(6.2)	0.157
Smoke	26 (24.5)	8(19.5)	18(27.7)	0.366
Alcohol abuse	13 (12.3)	4(9.8)	9(13.8)	0.762
C3 level, mg/mL	0.87 ± 0.2	1.06 ± 0.19	0.75 ± 0.14	< 0.001
C4 level, mg/mL	0.23 ± 0.1	0.25 ± 0.07	0.21 ± 0.06	0.003
Albumin level, g/dL	38.1 ± 7.5	37.6 ± 8.8	38.3 ± 6.6	0.634
Total bilirubin, μmol/L	10.1 ± 4.7	10.7 ± 5.1	9.6 ± 4.4	0.191
ASA grade				0.118
I + II	85 (80.2)	36(87.8)	49(75.4)	
≥ III	21 (19.8)	5(12.2)	16(24.6)	
Tumor location				0.187
GEJ	5(4.7)	4(9.8)	1(1.5)	
Upper 1/3	27(25.5)	8(19.5)	19(29.2)	
Middle 1/3	28(26.4)	12(29.3)	16(24.6)	
Lower 1/3	46(43.4)	17(41.4)	29(44.7)	
Depth of invasion				0.518
pT1	8 (7.6)	4(9.8)	4(6.2)	
pT2	11 (10.3)	5(12.2)	6(9.2)	
pT3	9 (8.5)	5(12.2)	4(6.2)	
pT4	78 (73.6)	27(65.8)	51(78.4)	
Lymph nodes metastasis				0.474
pN0	32 (30.2)	13(31.7)	19(29.2)	
pN1	14 (13.2)	6(14.6)	8(12.3)	
pN2	20 (18.9)	10(24.4)	10(15.4)	
pN3	40 (37.7)	12(29.3)	28(43.1)	
Histopathological type				*0.046*
Well diff.	7 (6.6)	3(7.3)	4(6.2)	
Moderate diff.	19 (17.9)	12(29.3)	7(10.8)	
Poor diff.	80 (75.5)	26(63.4)	54(83.0)	
Pathological Stage (AJCC 7th ed.)				0.414
I	12 (11.3)	7(17.1)	5(7.7)	
II	25 (23.6)	8(19.5)	16(24.6)	
III	55 (51.9)	22(53.7)	34(52.3)	
IV	14 (13.2)	4(9.8)	10(15.4)	
Neo-adjuvant chemotherapy	3(2.8)	1(2.4)	2(3.1)	0.847
Adjuvant therapy	78(73.6)	28(68.3)	50(76.9)	0.326

Data present with mean ± SD or number (percentage). Cut-off value for Low/High C3 group is 1.0 IHC C3 score. **Abbreviations:** *BMI*, body mass index; *HTN*, hypertension; *DM*, diabetes mellitus; *ASA*, American society of anesthesia; diff., differentiation.

### Complement C3 were highly expressed in primary GC tissues

**Fig. 2:**
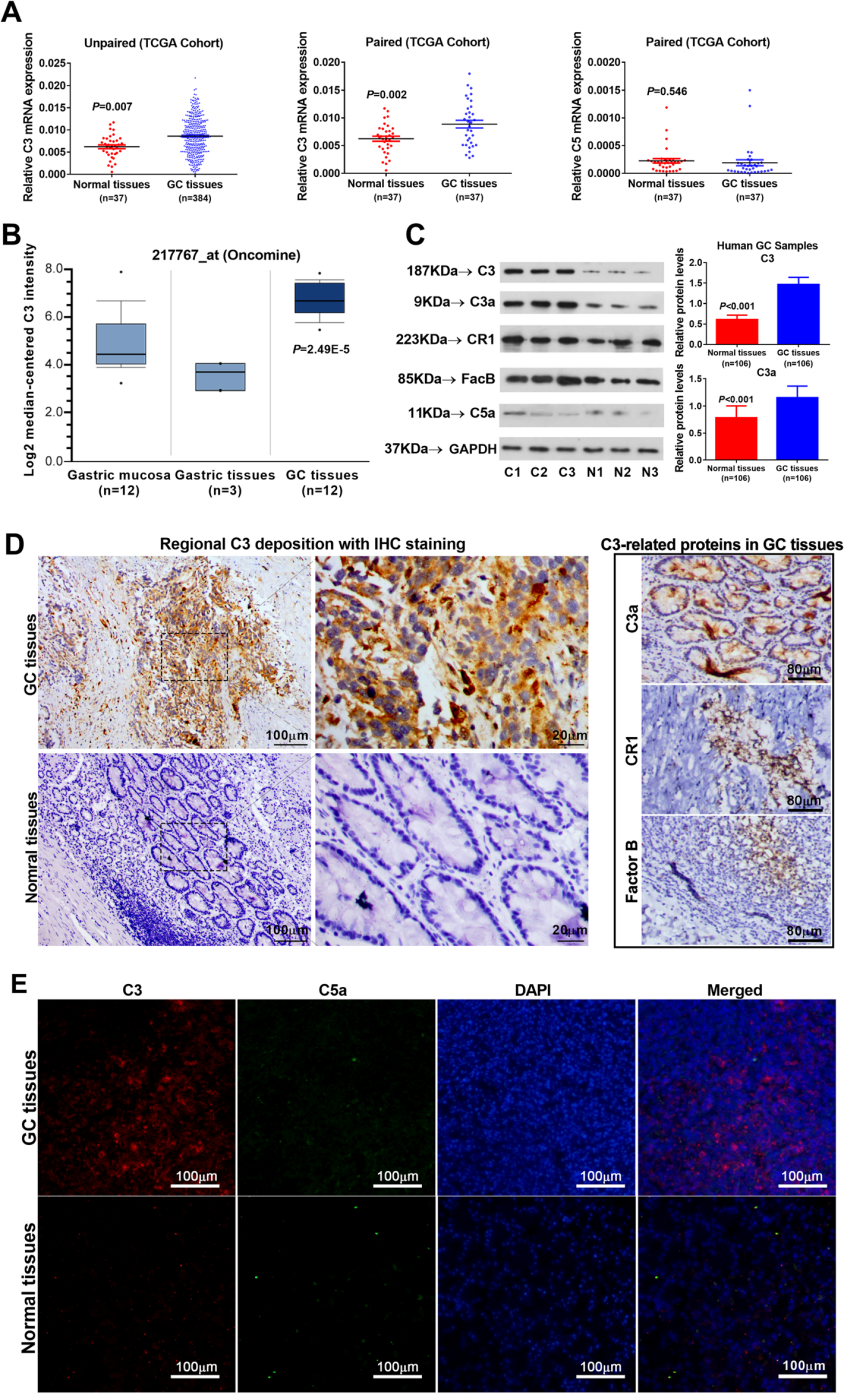
Increased expression of C3 in GC tissues. **a** Analysis of C3 expression in unpaired GC tissues and normal tissues in TCGA cohort (*P <* 0.001, left panel), C3 expression in paired GC and adjacent normal tissues (*n* = 37) in TCGA cohort (*P <* 0.001, middle panel), with C5 expression in the paired cohort (*P* = 0.546, right panel); **b** C3 expression in Gastric tissues according to Oncomine dataset (*P =* 2.49E-5; Reporter ID: 217767_at); **c** The protein levels of complement C3 and its effectors detected with western blot method in GC tissues and respective adjacent normal tissues (left panel; n = 3, left panel), with relative protein levels of C3 and C3a (right panel; *n* = 106, *P <* 0.001 vs normal tissues); Deposition of C3, C3a, C5a and the presence of CR1 and factor B in GC tissues were measured with IHC (**d**) and IFC (**e**) staining methods, with normal tissues utilized as control. Representative images of *n* = 5 independent experiments

In the bioinformatics TCGA cohort, the overall mRNA levels of C3 expressed in tumor tissues were markedly upregulated compared with normal gastric tissues (*P =* 0.007; Fig. 2a, left panel). The C3 upregulation was further validated in paired tumor and adjacent normal tissues (*P =* 0.002; Fig. 2a, middle panel); however, the C5 expression was not significantly different between paired samples (*P =* 0.546; Fig. 2a, right panel). In the Oncomine cohort, the C3 deposition was significantly enhanced in GC tissues compared with gastric mucosa or adjacent normal tissues (*P <* 0.001; Fig. 2b). Afterward, we analyzed the expression of C3 and other C3-related components in the paired GC and normal tissues from enrolled subjects. The protein levels of both C3 and C3a in GC tissues were highly increased compared with adjacent normal tissues (*P <* 0.001; Fig. 2c), with no significance observed for C5a, CR1 or fB levels. Moreover, regional depositions of C3 and C3a in GC tissues were markedly enhanced compared with C5a and other complement proteins (Fig. 2d&E).

### C3 deposition was associated with systemic complement depletion

**Fig. 3:**
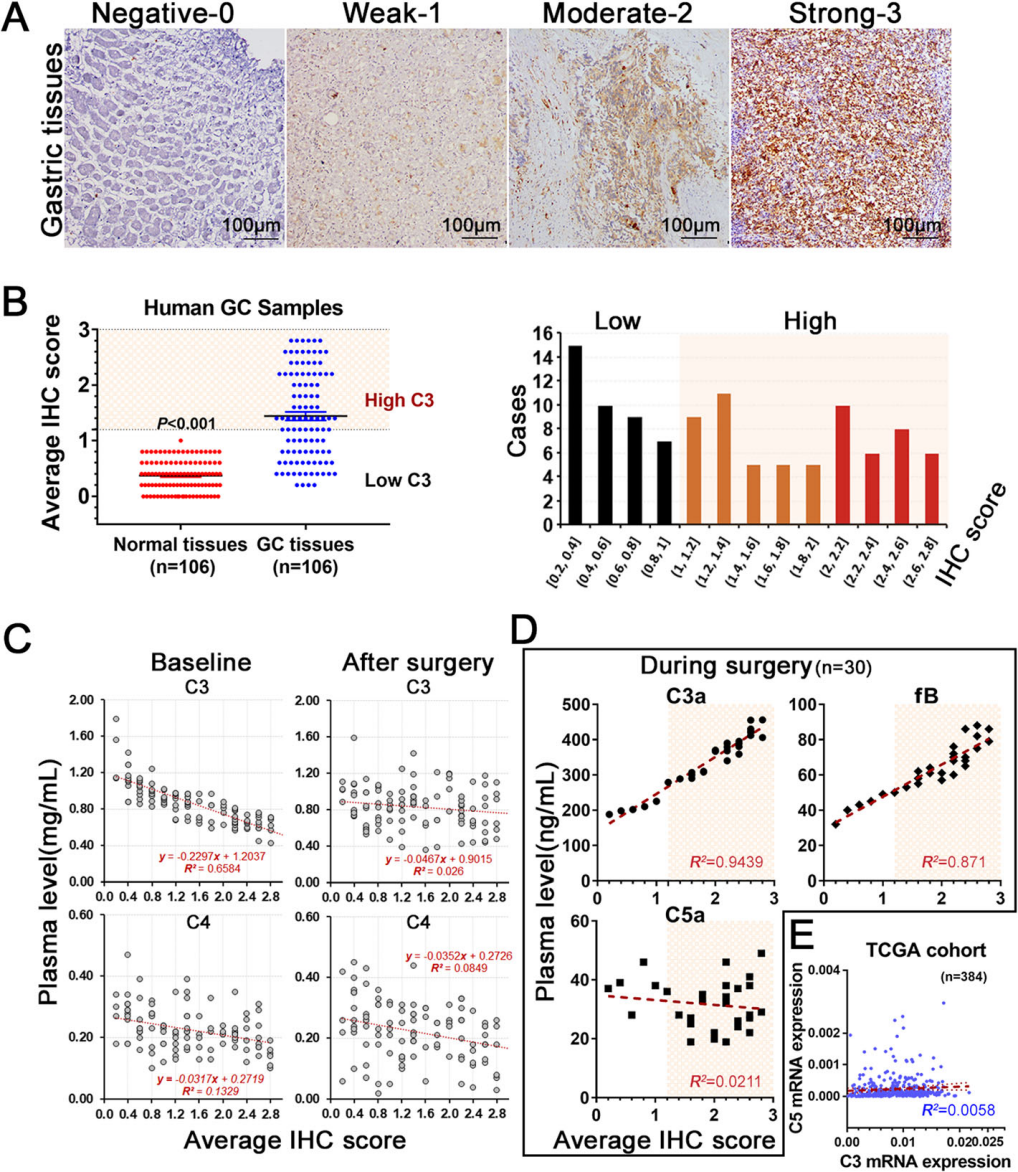
C3 deposition in GC tissues was associated with systemic Complement depletion. Regional deposition of C3 in GC tissues can be evaluated by the intensity of IHC staining (**a**), with score 0–3 for negative, weak, moderate and strong staining respectively. The average score of C3 deposition was calculated by five independent high-power fields of IHC section from each patient. The average score in primary GC tissues was much higher than that in adjacent normal tissues (**b**, left panel). Additionally, the case distribution based on C3 score presented as low (*n* = 41) and high C3 deposition (*n* = 35 as high and *n* = 30 as severe) in GC tissues (**b** right panel). The filling colors were black, brown and red for low, high and severe IHC scores, respectively. Linear relationship between average IHC C3 score and plasma levels of C3 and C4 at baseline and surgery was investigated (**c**). The relationship between IHC score and intraoperative plasma levels of C3a (*P <* 0.001), fB (*P <* 0.001) and C5a (*P =* 0.444) was explored using ELISA method (**d**, n = 30). The mRNA expression of C3 in GC tissues and C5 in the peripheral blood were extracted from TCGA database to assess their relationship, with no correlation found (**e**; *P =* 0.137, *n* = 384)

We employed the average score of C3 with IHC method (Fig. 3a) and divided patients into low and high C3 deposition groups with a cutoff value of 1.0 (Fig. 3b, left panel). We found that all patients were distributed into three subsets as trisection of IHC C3 scores, with 1:2 ratio for low and high C3 groups (Fig. 3b, right panel).

We investigated the relationship between localized C3 deposition and plasma levels including complement C3 and effectors at baseline, during surgery and after surgery, respectively. The linear regression results showed that average IHC C3 score was negatively correlated with systemic C3 level at baseline (***r***^***2***^ = 0.658, *P <* 0.001) and positively correlated with systemic C3a (***r***^***2***^ = 0.944, *P <* 0.001; Fig. 3c) and fB (***r***^***2***^ = 0.871, *P <* 0.001; Fig. 3d) levels during surgery. However, it was not associated with either C4 or C5a plasma level in the current cohort. The further external validation using TCGA cohort showed a non-correlation between localized C3 and plasma C5 expression in GC patients (*P =* 0.137; Fig. 3e).

### Enhanced C3 deposition predicted poor oncological outcomes

**Fig. 4:**
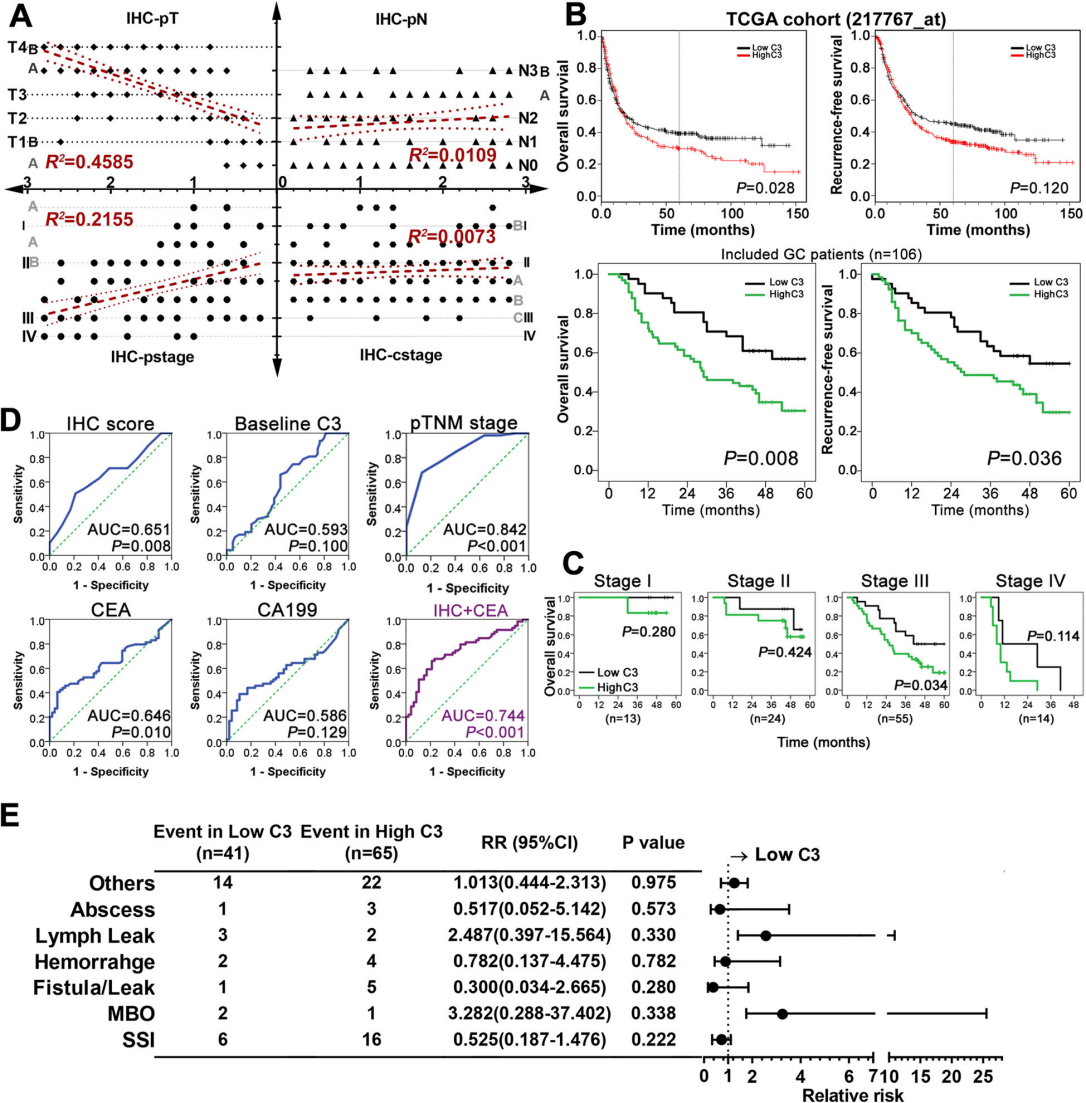
Enhanced deposition of C3 in GC tissues predicts advanced tumor stage and poor prognosis. **a** Regional deposition of C3 in GC tissues was strongly associated with advanced T stage and TNM stage (*P <* 0.001); however, it was not correlated with pathological N stage and clinical TNM stage in the current GC cohort (*P* > 0.05); **b** The 5-year overall survival and disease-free survival curves based on C3 deposition in enrolled subjects and TCGA samples; **c** The overall survival differences according to various tumor stage in our dataset; **d** The ROC curves of oncologic outcome (cancer-related death) based on C3 deposition, baseline C3 depletion, pathologic TNM stage, tumor markers (CEA and CA19–9) and combined factors (C3 deposition plus CEA); **e** Forest plot of short-term surgical outcomes (postoperative morbidities), with relative risk (RR) compared between the two groups

First, we explored the correlation between C3 deposition and tumor stage (Fig. 4 a). The findings indicated that it was positively correlated with pathological T (***r***^***2***^ = 0.459, *P <* 0.001) and TNM stages (***r***^***2***^ = 0.2155, *P <* 0.001), but not related to pathological N stage (*P =* 0.287) or clinical TNM stage (*P =* 0.383).

**Fig. 5:**
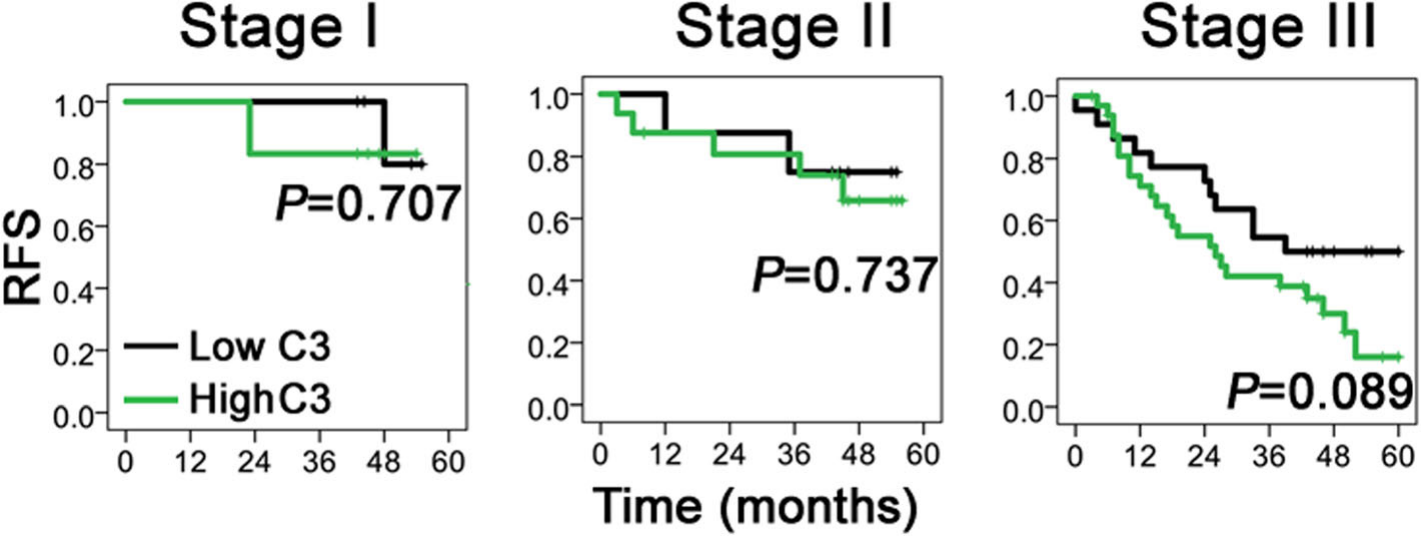
The long-term recurrence-free survival of GC patients with low or high C3 deposition in each tumor stage. Kaplan-Meier curves were employed to compare survival significance among stage I, II and III. Of note, patients with stage IV were excluded from such comparison

Second, we reviewed the long-term outcomes of GC patients from the TCGA dataset. We found that patients with high C3 expression in GC tissues had poorer overall survival (OS; Fig. 4b, left upper quadrant) and recurrence-free survival (RFS; Fig. 4b, right upper quadrant) than those with low C3 expression, with a survival significance observed in OS (*P =* 0.028). Subsequently, we compared the 5-year survival outcomes with our data (Fig. 4bb, left and right lower quadrants) and confirmed that high C3 deposition was a predictive factor of poor OS (*P =* 0.008) and RFS (*P =* 0.036). The 5-year OS and RFS rates were 52.6 and 50.7% in the low C3 group and 29.7 and 28.2% in the high C3 group, respectively. By further subgroup survival analyses based on tumor stage (Fig. 4c), we detected a survival significance of C3 deposition in stage III patients (*P =* 0.034), with no significance observed in other stages (*P* > 0.05). The results for RFS were not significant in each stage (Fig. 5).

Third, we performed a ROC curve analysis including IHC C3 score, plasma C3 level at baseline, pathological stage and two tumor markers to determine the prognostic value for tumor-related death (Fig. 4d). The findings indicated that both IHC C3 score (AUC = 0.651) and serum CEA level (AUC = 0.646) were valuable in predicting oncologic outcome, but inferior to pathological tumor stage (AUC = 0.842). We combined both useful parameters and obtained a better value (AUC = 0.744) that was comparable to tumor stage. The ROC analyses revealed that optimal cut-off values were 1.4 for IHC C3 score and 4.2 ng/ml for CEA level. We also compared the incidence of postoperative morbidity between both groups (Fig. 4e), which suggested that the localized C3 deposition was not significantly associated with any morbidities after surgery (P > 0.05). Of note, the relative risk (RR) of surgical site infection (RR, 0.525; 95% confidential interval [CI], 0.187–1.476) and anastomotic leak (RR, 0.300; 95%CI, 0.034–2.665) was both reduced in the low C3 group compared with the high C3 group.

At last, we determined the prognostic value of localized C3 deposition by using univariate and multivariate Cox regression analyses against 5-year OS (Table 2). We verified that high C3 deposition in GC tissues (odds ratio [OR], 1.848; 95%CI, 1.015–3.363; *P* = 0.045), along with advanced tumor stages (stage III and IV; OR, 2.609; 95%, 1.725–4.194; *P* < 0.001), depleted plasma C3 level (< 0.75 mg/ml; OR, 1.801; 95%CI, 1.049–3.090; *P* = 0.033) and any morbidities after surgery (OR, 2.770; 95%, 1.446–5.305; *P* = 0.002), were independent factors for poor 5-year OS in GC patients.

**Table 2:** Univariate and Multivariate cox-regression analyses of prognostic factors for gastric cancer

Factor	Number	5-year OS				
		Univariate analysis			Multivariate regression ^a^	
		Cumulative Rate(%)	OR(95%CI)	*P*-value	OR(95%CI)	*P*-value
Age (≥65:< 65 years)	24:82	34.0:40.5	0.948(0.520–1.727)	0.978		
BMI (≥25.0:< 25.0 kg/m^2^)	13:93	35.9:39.5	0.916(0416–2.018)	0.842		
Gender (Male:Female)	65:41	40.4:37.6	1.355(0.807–2.275)	0.230		
Blood Type (A:AB:B:O)	43:10:22:30	44.4:15.6:46.4:34.2	1.092(0.496–2.405)	0.936		
Tumor location (GEJ:Upper:Middle:Lower *third*)	5:27:28:46	26.7:20.2:46.4:51:1	0.988(0.915–1.067)	0.705		
Surgical resection (Proximal:Distal:Total)	13:45:48	35.9:46.6:32.4	1.143(0.776–1.682)	0.538		
TNM stage (I + II:III + IV)	37:69	67.9:23.4	3.336(2.205–5.049)	0.001	2.609(1.725–4.194)	< 0.001
Histology (Well:Moderate:Poor)	7:19:80	33.3:46.4:40.3	1.154(0.761–1.750)	0.128	1.049(0.653–1.686)	0.844
IHC C3 score (Low:High))	41:65	52.6:29.7	2.109(1.198–3.714)	0.005	1.848(1.015–3.363)	0.045
Plasma C3 (Low:Normal)	40:66	22.9:47.2	1.730(1.034–2.897)	0.062	1.801(1.049–3.090)	0.033
CEA (Normal:Elevated)	81:25	48.5:7.4	2.673(1.556–4.592)	0.005	1.225(0.680–2.208)	0.500
Morbidity ^b^ (Yes:No)	59:47	72.3:5.2	4.799(2.669–8.628)	< 0.001	2.770(1.446–5.305)	0.002

^a^Those factors were qualified from the univariate analysis of overall survival, which their *P* values were less than 0.20. ^b^ Any postoperative complication was considered as morbidity and pooled together for survival comparison. Abbreviations: OS, overall survival; DFS, disease-free survival; OR, odds ratio; CI, confidential intervals; BMI, body mass index; SLRC, SMA-guided laparoscopic right hemicolectomy; CLRC, conventional laparoscopic right hemicolectomy.

### High expression of C3 promoted tumor progression in GC cell lines

**Fig. 6:**
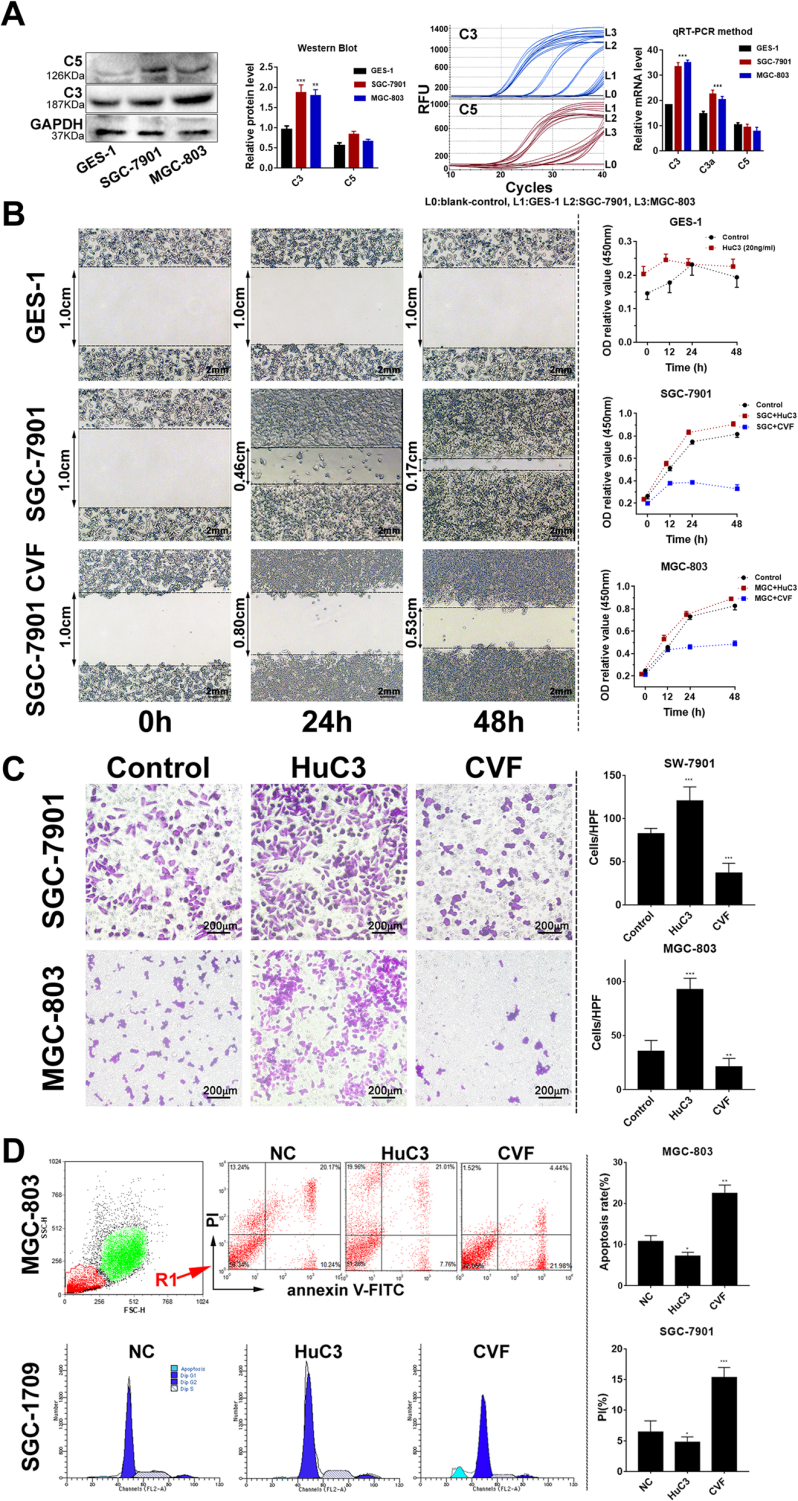
Enhanced expression of C3 promoted tumor progression in GC cell lines. **a** Overexpression of C3 in human GC cell lines (SGC-7901 and MGC-803) detected by western blot and qRT-PCR methods, with normal gastric cell line (GES-1) as control; **b** Exogenous C3 stimulation promoted the migration of GC cells (left panel). The time-dependent cell proliferation was inhibited by CVF in both GC cell lines (right panel); **c** Inhibition of C3 activation with CVF significantly inhibited the invasion of GC cells; **d** Flow cytometry study to investigate the apoptosis rate of GC cells. Early stage of apoptosis was detected by propidium iodide (PI) and annexin V-fluorescein isothiocyanate (V-FITC) dual staining assay. 20,000 cells per sample in all in vitro assays, representative sparklines and histograms (right panel) of *n* = 5 independent experiments

We examined RNA and protein expression of C3 and complement effectors in GC (SGC-7901 and MGC-803) and gastric mucosa (GES-1) cell lines (Fig. 6a). We found that both C3 and C3a were highly expressed in SGC-7901 and MGC-803 compared with GES-1; whereas C5 was similarly expressed across those cell lines. Besides, we observed a significantly decreased cell migration in CVF-treated SGC-7901 after 48 h of culturing (Fig. 6b, left panel). Exogenous C3 treatment could enhance cell proliferation in both SGC-7901 and MGC-803, but quickly shut down such growth once CVF was added into the C3-contained culture medium (Fig. 6b, right panel). Additional invasion experiments indicated that exogenous C3 could promote invasion capacity, which could be markedly depressed by CVF (Fig. 6c).

Next, we performed flow cytometric analysis of cell cycle and apoptosis (Fig. 6d). Exogenous C3 caused a dramatic decrease of apoptosis in MGC-803 cells compared with NC (10.8% vs. 7.3%, *P =* 0.0462). The use of CVF in the CM resulted in a reverse increase of apoptosis compared with NC (22.5% vs. 7.3%, *P <* 0.001). Meanwhile, the cell cycle study in SGC-7901 also confirmed an increased percentage of cells in S phase from C3 treatment (32.6% vs. 19.7%, *P =* 0.013) and an enhanced population in apoptotic phase from CVF interference (15.3% vs. 6.4%, *P =* 0.003).

### JAK2/STAT3 signaling pathway was responsible for downstream regulation of C3 deposition

**Fig. 7:**
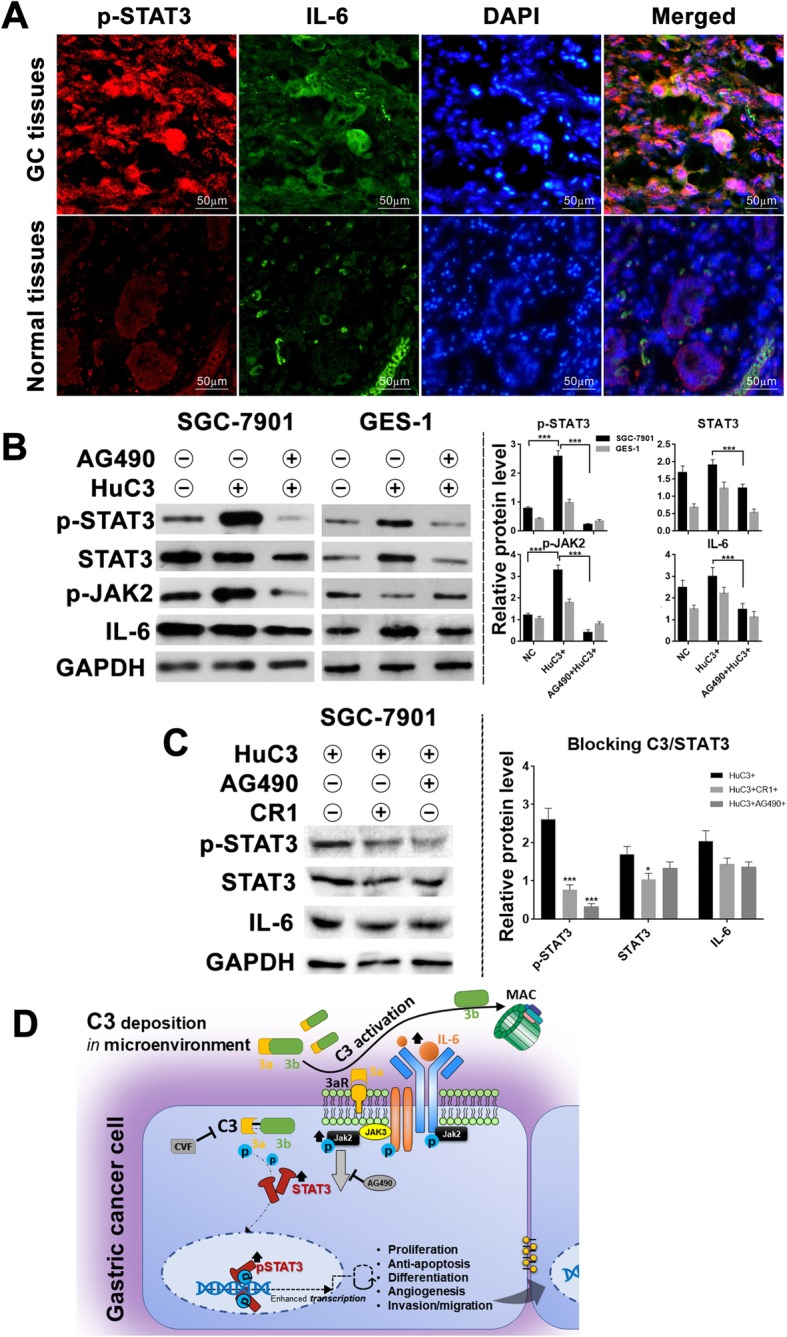
JAK2/STAT3 signaling pathway was related to C3 deposition in GC tissues and C3-induced oncoprogression. **a** Typical expression of p-STAT3 and IL-6 in GC and adjacent normal tissues (IFC method), which indicates an up-regulation of STAT3 signaling in GC patients (representative images of n = 5 independent experiments); **b** Levels of JAK2/STAT3-related proteins (IL-6, p-JAK2, p-STAT3 and STAT3) were detected on SGC-7901 and normal GES-1 cell line with WB method (left panel). The STAT3 signaling was highly activated with exogenous C3 treatment, and greatly inhibited when JAK2-blocker (AG490) was pre-incubated with C3 (right panel); **c** Levels of p-STAT3 and IL-6 in C3-antagonist pre-treated GC cells (upper panel). The JAK2/STAT3 signaling remained activated but weakened under blockage of C3 signaling with CR1 compared with blockage of JAK2 with AG490 (lower panel). 20,000 cells per sample in all in vitro assays, representative histograms (right panel) of n = 5 independent experiments; **d** A proposed model for the underlying mechanism of C3/JAK2/STAT3 signaling pathway participating in the pathogenesis of GC. Abbreviations: 3aR, Complement C3a receptor; MAC, membrane attack complex; CVF, cobra venom factor

We detected the activation of JAK2/STAT3 axis in human GC tissues first. Expression of both STAT3 phosphorylation (p-STAT3) and IL-6 were significantly enhanced in GC tissues compared to adjacent normal tissues (Fig. 7xa). A similar result was observed in comparing SGC-7901 with GES-1 in vitro. Afterward, we treated SGC-7901 with exogenous C3 and detected increased expression of p-STAT3 and p-JAK2 (Fig. 7b). However, pre-incubation of cells with AG490 and exogenous C3 significantly blocked the C3-induced increases in JAK2/STAT3 phosphorylation, which indicated that C3 might work as an upstream regulation of JAK2/STAT3 activation. We employed CR1 to block exogenous activation of C3 and detected a weakened expression of p-STAT3 and IL-6 compared with AG490-treated cancer cells (Fig. 7c). These data indicated that localized activation and deposition of C3 may play a role in tumor growth and metastasis by potentiating JAK2/STAT3 activation (Fig. 7d).

## Discussion

Complement C3 is the central molecular for complement activation cascades. Following C3 activation, several effectors, such as C3a and C5a, commonly act as potent pro-inflammatory anaphylatoxins stimulating recruitment and activation of immune cells, and especially, leading to formation of regional inflammation and cellular lysis [29]. However, C3 might play a double-edged role in the tumor microenvironment. Several studies have shown that it could facilitate cellular proliferation and regeneration by dysregulating mitogenic signaling pathways, sustaining angiogenesis and oncogenesis [30, 31].

The internal synthesis and secretion of C3 were observed in various immune cells and GC cells [32–34]. Our study confirmed that both SGC-7901 and MGC-803 cell lines produced intracellular C3. It suggested that C3 might be expressed by the neoplastic epithelia as a component of tumor pathology, and thereby contribute to local immune responses. We proposed that intracellular C3 activation provided essential signals to initiate the JAK2/STAT3 pathway and to subsequently promote cell proliferation and migration. That could explain why JAK2/STAT3 activation was weakened rather than totally shut down when exogenous C3 treatment was inhibited with CR1.

Activated STAT3 protein acts as a transcriptional factor to regulate cell proliferation, apoptosis, angiogenesis, tumor invasion and metastasis [35]. A meta-analysis confirmed that high expression of p-STAT3 was associated with poor prognosis of GC [36]. In addition, clinical usage of a STAT3-regulated microRNA signature showed a prognostic potential in early GC stratification [37]. Our study indicated a direct relationship between local C3 and JAK2/STAT3 pathway activation in GC patients and identified a promotional effect of C3 on STAT3 activation through an inflammatory cytokine, which was consistent with previous reports [38, 39]. Moreover, complement-triggered phosphoinositide 3-kinase pathway has been confirmed in the pathogenesis of GC [40, 41]. A variety of complement inhibitors targeting the mediators of complement activation are proposed to have great potential in cancer therapy [12, 13, 42].

The current GC staging system provides incomplete prognostic information [43–45]. Consequently, novel immune signatures, such as immunoscore and tumor-infiltrating neutrophils, were recently proposed for GC classification and prognosis prediction [46, 47]. The usage of such signatures achieved an improvement in prediction of chemotherapeutic or survival benefits for GC patients. Our method, combining C3 activation with a tumor marker (serum CEA), obtained a comparable prognostic value for pathological TNM staging which is supported using the IHC C3 score as an immune signature for GC classification.

Our present study had limitations. First, it was a single center cohort study with limited generalizability. No healthy control group was included for serum complement level comparison. Second, the small sample size may conceal differences in relevant surgical and oncological outcomes, such as incidence of readmission and morbidity, disease-free and progression-free survivals. Additional validation by a cohort from another center would be helpful to verify our findings. At last, a concrete mechanism of C3/JAK2/STAT3 signaling was not determined due to limited in vitro experiments and lack of animal study. Several complement receptors and regulators (CD35, CD46, CD55, CD59, CD88, etc.), which have better control local C3 activation at the cell membrane, would be detected in more GC cell lines as our future works. Besides, a further investigation of STAT3-related factors in responsive to localized C3 deposition would be indispensable to validate our results.

## Conclusions

Complement C3 activation, characterized by localized deposition of C3 and its effectors together with reduced plasma C3 levels, appears to contribute the tumor progression and poor prognosis in human GC. Enhanced C3 deposition and activation in the microenvironment of GC tissues correlated with local inflammation and tumor cell invasion. Importantly, localized C3 deposition activated the JAK2/STAT3 signaling pathway which we propose would cause inferior oncologic outcomes. The potential of using C3 deposition as an immune signature in predicting GC recurrence and survival is now demonstrated but needs further validation.

## Supplementary information

**Additional file 1: Table S1.** Primers used in qRT-PCR experiments.

**Additional file 2: Table S2.** Surgical outcomes of patients with gastric cancer.

## Data Availability

The datasets used and analyzed during the current study are available from the corresponding author on reasonable request.

## Abbreviations

AUC: Area under the curve
CI: Confidential interval
CR1: Complement receptor
CVF: Cobra venom factor
ELISA: Enzyme-linked immunosorbent assay
FB: Factor B
FBS: Fetal bovine serum
GC: Gastric cancer
IFC: Immunofluorescence and confocal analysis
IHC: Immunohistochemistry
JAK2/STAT3: Janus kinase 2/signal transducers and activators of transcription
NC: Normal control
OR: Odds ratio
OS: Overall survival
PVDF: Polyvinylidene fluoride
QRT-PCR: Quantitative real-time polymerase chain reaction
RFS: Recurrence-free survival
ROC: Receiver operating characteristics
TCGA: The Cancer Genome Atlas
WB: Western blotting
