# Supplementary material for: Complement C3 overexpression activates JAK2/STAT3 pathway and correlates with gastric cancer progression
Source: J Exp Clin Cancer Res. 2020 Jan 13;39:9. doi: 10.1186/s13046-019-1514-3 (PMC6956509; doi:10.1186/s13046-019-1514-3)
Supplement: Supplementary file 1 — Additional file 1: Table S1. Primers used in qRT-PCR experiments. [file 13046_2019_1514_MOESM1_ESM.docx]

Table S1. Primers used in qRT-PCR experiments

| **Target gene** | **Forward primer（5’-3’）** | **Reverse primer（5’-3’）** | **Accession ID** | **Max BLAST Score** |
| --- | --- | --- | --- | --- |
| C3, 187bp | TCACCGTCAACCACAAAGCTGCTACC | TTTCATAGTAGGCTCGGATCTTCCA | [NM_000064.4](https://www.ncbi.nlm.nih.gov/entrez/viewer.fcgi?db=nucleotide&id=1653961407) | 36.2 |
| C3a, 102bp | AGCTGAAGCACCTCATTGTGA | TCTGGGTGTACCCCTTCTTGA | [NM_001037236](http://www.ncbi.nlm.nih.gov/entrez/viewer.fcgi?val=NM_001037236) | 41.0 |
| C5, 188bp | GTTGAAGCCCGAGAGAACAG | AGGGAAAGAGCATACGCAAGA | [NM_001317163.2](https://www.ncbi.nlm.nih.gov/nucleotide/NM_001317163.2?report=genbank&log$=nucltop&blast_rank=1&RID=R1B89FNK015) | 40.1 |
| GAPDH, 111bp | AAGAAGGTGGTGAAGCAGG | GAAGGTGGAAGAGTGGGAGT | [NM_001357943.2](https://www.ncbi.nlm.nih.gov/nucleotide/NM_001357943.2?report=genbank&log$=nucltop&blast_rank=25&RID=R1BXWSC8014) | 38.2 |
| All primers were synthesized at VCU DNA core facility (Shanghai, China). Primers tested with BLAST analysis and used when BLAST score ≥35.0. | | | | |
