# Supplementary material for: Complement C3 overexpression activates JAK2/STAT3 pathway and correlates with gastric cancer progression
Source: J Exp Clin Cancer Res. 2020 Jan 13;39:9. doi: 10.1186/s13046-019-1514-3 (PMC6956509; doi:10.1186/s13046-019-1514-3)
Supplement: Supplementary file 2 — Additional file 2: Table S2. Surgical outcomes of patients with gastric cancer. [file 13046_2019_1514_MOESM2_ESM.docx]

| **Table S2.** Surgical outcomes of patients with gastric cancer | | | |
| --- | --- | --- | --- |
|  | **Low C3 group**  **(n=41)** | **High C3 group**  **(n=65)** | ***P*-value** |
| Surgical approach |  |  | *0.832* |
| Open | 38(92.7) | 60(92.3) |  |
| Laparoscopic | 3(7.3) | 5(7.7) |  |
| Operation time, min | 260.9±77.9 | 289.8±77.3 | 0.065 |
| Estimated bleeding, ml | 284.2±419.9 (150) | 216.2±157.0 (200) | 0.243 |
| Fluid intake, ml | 4162.2±1303.1 | 3932.3±1032.1 | 0.316 |
| Fluid output, ml | 1206.6±1121.4 | 1196.0±754.8 | 0.954 |
| Blood transfusion |  |  | 0.832 |
| None | 31(75.6) | 53(81.5) |  |
| <500ml | 3(7.3) | 5(7.7) |  |
| ≥500 and ≤1000ml | 6(14.6) | 6(9.2) |  |
| >1000ml | 1(2.4) | 1(1.5) |  |
| Tumor location |  |  | 0.187 |
| GEJ | 4(9.8) | 1(1.5) |  |
| Upper 1/3 | 8(19.5) | 19(29.2) |  |
| Middle 1/3 | 12(29.3) | 16(24.6) |  |
| Lower 1/3 | 17(41.4) | 29(44.7) |  |
| Gastrectomy |  |  | 0.833 |
| Proximal | 5(12.2) | 8(12.3) |  |
| Distal | 16(39.0) | 29(44.6) |  |
| Total | 20(48.8) | 28(43.1) |  |
| Lymphadenectomy |  |  | 0.997 |
| D_1_ | 3(7.3) | 5(7.7) |  |
| D_2_ | 29(70.7) | 46(70.7) |  |
| D_2_ plus ^a^ | 9(22.0) | 14(21.6) |  |
| Harvest LNs | 37.6±16.3 | 40.2±20.3 | 0.496 |
| Metastatic LNs | 7.1±10.3 | 8.8±11.9 | 0.441 |
| Reconstruction |  |  | 0.936 |
| Billroth I | 1(2.4) | 1(1.5) |  |
| Billroth II | 4(9.8) | 7(10.8) |  |
| Roux-en-Y^b^ | 36(87.8) | 57(87.7) |  |
| LOS, days | 19.5±8.6 | 20.7±6.9 | 0.459 |
| Postoperative LOS | 11.2±7.5 | 11.0±5.5 | 0.893 |
| Hospital expense, $ | 9960.2±4853.6 |  | 0.189 |
| Postoperative morbidity | 16(39.0) | 31(47.7) | 0.382 |
| 30-day operative mortality | 0 | 0 | - |
| Data present as mean±SD or number (percentage). **Abbreviations:** GEJ, gastro-esophageal junction; LNs, lymph nodes; LOS, length of stay. ^a^ D2 plus means extended lymphadenectomy beyond D2 stations during gastrectomy. ^b^ including classical and modified en-Y anastomosis techniques. “-“ indicates unavailable value. | | | |
